# Menopausal Hormone Therapy in Breast Cancer Survivors

**DOI:** 10.3390/cancers16193267

**Published:** 2024-09-26

**Authors:** Rose Culhane, Alexandra M. Zaborowski, Arnold D. K. Hill

**Affiliations:** Department of Breast Surgery, Beaumont Hospital, P.O. Box 1297 Dublin 9, Ireland

**Keywords:** menopausal hormone therapy, breast cancer, recurrence

## Abstract

**Simple Summary:**

Menopause remains a complex challenge after breast cancer. Menopausal symptoms, whether iatrogenic or premature due to cancer treatment, significantly affect health and quality of life. Menopausal hormone therapy is currently deemed unsafe in hormone-sensitive breast cancer. Unfortunately, the alternative non-hormonal therapies are not as effective in controlling menopausal symptoms. The aim of our narrative review was to determine the risk of recurrence of breast cancer in breast cancer survivors taking menopausal hormone therapy. Data on this topic are of low quality and outdated. This review highlights the need for future research to help navigate this difficult path.

**Abstract:**

Menopausal symptoms negatively impact quality of life in breast cancer survivors. The paucity of data on the impact of Menopausal Hormone Therapy (MHT) on oncological outcomes in these patients limits informed clinical discussion. Defining the risk of cancer recurrence with MHT is central to the appraisal of risk/benefit, particularly with low-risk disease (based on genomic profile). The aim of this review is to summarize the current data evaluating MHT in breast cancer patients. A systematic review of the literature was performed to evaluate the impact of MHT on oncological outcomes in breast cancer survivors. Three major databases (PubMed, EMBASE and Medline) were searched. The review included all prospective studies published in English. Four randomized control trials and four non-randomized prospective studies were identified. An increase in breast cancer recurrence with MHT was observed in the early randomized trials whilst no increased risk of recurrence was reported in the observational studies. There remains a need to quantify MHT-related recurrence risk in patients with molecularly favorable disease.

## 1. Introduction

The role of menopausal hormone therapy (MHT) in breast cancer survivors remains undefined. Concerns regarding oncological safety, particularly in patients with hormone receptor-positive cancers, have led to physician and patient reluctance to prescribe and take MHT, respectively. Data are lacking and limited to a few studies. Furthermore, interpretation is hampered by limitations in methodology and length of follow up. MHT has the potential to significantly improve menopausal symptoms (vasomotor, urogenital and cognitive) and quality of life, as well as reduce the long-term adverse effects of estrogen deficiency such as cardiovascular disease and osteoporosis [1]. Breast cancer survivors frequently suffer from iatrogenic or premature menopause due to adjuvant chemotherapy or endocrine therapy [2]. An improvement in prognosis has been observed with escalation of adjuvant therapy. This has coincided with an increased burden of vasomotor symptoms, which have been demonstrated to have a detrimental impact on quality of life [3,4,5]. Importantly, iatrogenic side effects are strongly correlated to non-adherence to adjuvant therapy in postmenopausal patients [6,7]. Thus, controlling these troublesome symptoms remains a pressing issue [8]. Hesitancy to prescribe MHT in breast cancer survivors is mainly based on the results of a Swedish clinical trial conducted almost three decades ago [9]. Four randomized control trials have evaluated MHT in breast cancer survivors, all of which reported increased rates of disease recurrence with MHT [9,10,11,12]. However, recent observational studies have failed to identify a significant increase in disease recurrence. The aim of this review is to summarize the data on the impact of MHT on oncological outcomes in breast cancer survivors.

## 2. Materials and Methods

**Figure 1:**
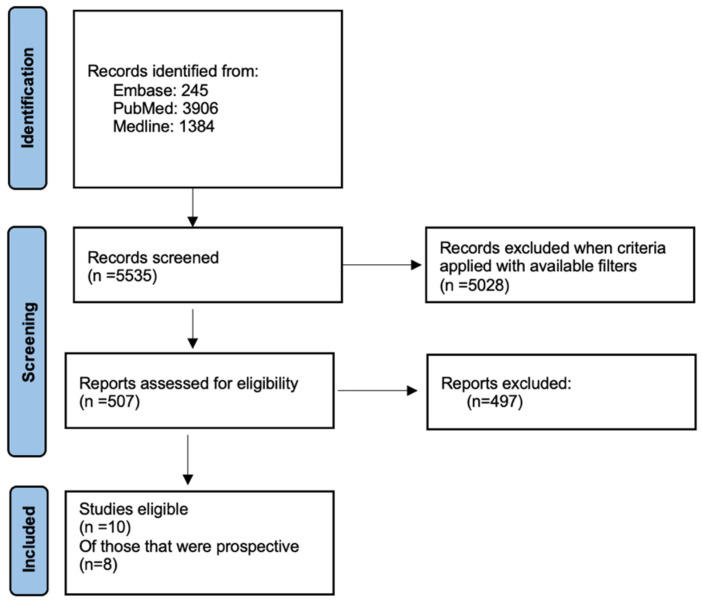
Flow chart illustrating screening and selection process.

The literature search was completed on three databases: PubMed, EMBASE and Medline as shown in Figure 1. The search strategy was conducted using the following combination of search terms: (“breast cancer” OR “breast neoplasm*” OR “breast tumour*” OR “breast tumor*” OR “breast carcinoma*”) AND (“hormone replacement therapy” OR HRT OR “estrogen replacement therapy” OR ERT OR tibolone OR “menopausal hormone therapy”) AND (recurrence OR “rate of recurrence” OR risk OR survivor* OR relapse). Criteria of inclusion were after 1990, in English, RCT/clinical trials and prospective cohort/observational studies, sample size greater than 30, studies involving adult patients, and studies including adults with previous breast cancer. Duplicates and retrospective studies were eliminated.

## 3. Results

### 3.1. Randomized Control Trials

Four randomized control trials (RCTs), as shown in Table 1, have evaluated the impact of MHT on oncological outcomes in breast cancer survivors [10,11,12,13]. Two trials used estrogen- and progestogen-based MHT, one used estrogen only and one used the synthetic steroid tibolone. Two Swedish RCTs conducted in the 1990s, the Hormonal Replacement therapy After Breast Cancer—Is It Safe? (HABITS) trial and the Stockholm trial, included patients with estrogen receptor positive and negative breast tumors. The larger HABITS trial randomized 442 patients to MHT or non-hormonal symptom management [9]. The MHT regimen included mainly estradiol with or without norethisterone acetate depending on the presence of a uterus. The Stockholm trial randomized 278 women to MHT or no hormonal treatment. Patients less than 55 years old received 2 mg of estradiol for 21 days and an additional 10 mg of medroxyprogesterone acetate for the last 10 days followed by 7 days with no treatment. Patients aged 55 years or above received 2 mg of estradiol for 84 days and an addition of 20 mg of MPA for the last 14 days followed by 7 days with no treatment. Participants who had undergone a hysterectomy received continuous 2 mg of estradiol valerate daily.

In 2002, the data of both trials were pooled, and an interim safety analysis was performed. A significantly increased risk of recurrence was observed with MHT (combined hazard ratio of 1.8) and both trials were stopped early in 2003 [14]. In the HABITS trial, the rate of breast cancer recurrence was significantly higher in the MHT arm (HR 3.3) [9]. This finding led to the discontinuation of the Stockholm trial. Interestingly, the Stockholm trial had an HR (hazard ratio) of 0.82 at the time of analysis. Both trials subsequently performed a longer-term analysis. The HABIT group followed their patients for another four years, which still showed an increased risk of recurrence in breast cancer survivors taking MHT with a hazard ratio of 2.4 [13]. At 10 years follow up, the Stockholm trial revealed no overall increased risk for breast cancer recurrence with a hazard ratio of 1.3; however, there was a significant increase in contralateral breast cancer [10]. Several noteworthy differences between both trials may in part explain why the results were so contradictory. Firstly, the use of progesterone was inconsistent between the two trials. In the HABITS trial, patients in the MHT arm were exposed to a higher potency of progesterone than the MHT group of the Stockholm trial. HABITS used a potent testosterone-like progestogen (norethisterone acetate), whilst Stockholm used a low-dose and naturally occurring progesterone medroxyprogesterone acetate. It is known that adding progesterone to an MHT regime does increase the risk of breast cancer, and that norethisterone acetate would cause more of an increased risk of breast cancer than medroxyprogesterone acetate would [15]. Furthermore, the HABITS trial had slightly more node-positive patients than the Stockholm trial (20% vs. 18%) and Stockholm had a larger amount of tamoxifen users (52% vs. 21%). The goal of the HABITS trial originally was to recruit 1300 women to exclude the possibility that 2 years of MHT conferred a relative risk of new breast cancer that exceeded 1.36; however, these numbers were not met during recruitment [13].

A smaller North American RCT included patients with ER (estrogen receptor)-negative tumors who were disease-free for at least 2 years and patients with unknown ER status who were disease-free for at least 10 years [11]. An estrogen-only MHT regimen was used; patients received 0.625 mg of Premarin on days 1–25 of each month. Progesterone was omitted and annual gynecological assessments were performed. Seventy-seven patients were randomized to MHT (*n* = 34) or no hormonal treatment (*n* = 43). At 5 years follow up, breast cancer recurrence occurred in two women in the MHT group and four women in the non-MHT group. A hazard ratio of 0.63 was calculated to conclude that there was a better outcome in the MHT group in terms of recurrence. This trial had aimed to recruit enough participants to have the power to detect a hazard ratio of 2.1 associated with taking MHT, but did not obtain the required number. A group of eligible women who declined to be a part of the trial were also followed up. Of those, 22 had taken MHT as prescribed by their own physicians. In combination with the non-participants, 2 of the 56 women taking MHT had breast cancer recurrence (3.6%) and 33 of the 243 not taking MHT had recurrence (13.5%). Overall, this small study suggested that MHT may be oncologically safe in hormone receptor-negative breast cancer survivors. A larger scale trial would be required to confirm these results.

The Liberate trial that recruited 3098 women with previous breast cancer was the only RCT that achieved its recruitment goal sufficient for statistical power. Rather than an estrogen/progesterone regimen, this trial looked at the risk of recurrence of breast cancer if tibolone was given to manage vasomotor symptoms [16]. Tibolone is a synthetic steroid with a pharmacological and clinical profile different from conventional steroids. Following oral administration, tibolone is rapidly metabolized into three compounds. Two of the metabolites (3α-OH-tibolone and 3β-OH-tibolone) have estrogenic-like activities, whereas the third metabolite (Δ 4-isomer of tibolone) has progestogenic and androgenic-like activities. It is effective in preventing bone loss and treating climacteric symptoms of menopause, without stimulating the endometrium [17]. After randomization, 1556 women received 2.5 mg of tibolone daily and 1542 received the placebo. Due to an excess trend of breast cancer recurrence in the tibolone group, the trial ended prematurely. After a median follow up of 3.1 years, there was breast cancer recurrence in 237 of 1552 (15.2%) of women in the tibolone group compared to 165 of 1542 (10.7%) in the placebo group. This yielded a hazard ratio of 1.40 [16].

A meta-analysis of all four trials by Poggio et al. reported an overall hazard ratio of 1.46 and a significant increased risk of recurrence of breast cancer with systemic MHT use [14]. Across the three larger randomized control trials that included ER-positive patients, MHT did increase the risk of overall breast cancer recurrence. Interestingly the Stockholm trial alone reported a non-significant increased risk [10,12,13]. Since these trials, there has been resistance from physicians prescribing MHT for breast cancer survivors. More recently, the UK HRT Trial failed to recruit 3000 women over 3–5 years [18]. The trial was closed at the end of January 2004 owing to recruitment being slower than expected. To our knowledge, this was the last attempt at an RCT.

### 3.2. Prospective Observational Cohort Studies

On the contrary, there have been several observational cohort studies published to suggest that MHT is not associated with an increase in the risk of recurrence in breast cancer [19]. However, the quality of these reports is questionable in comparison to the randomized control trials and the majority are retrospective, but still hold some value. Here, we will summarize the outcome of prospective studies available beyond the clinical trials already discussed.

Marttunen et al. published the results of a prospective study that evaluated 131 postmenopausal breast cancer survivors suffering with menopausal symptoms [20]. The 131 women were given the option of MHT or no MHT. Forty-three women refused and eighty-eight commenced MHT. Hysterectomized women received estradiol either orally or trans dermally and non-hysterectomized women received the same but in combination with a 10-day course of oral medroxyprogesterone acetate (MPA) at 4-week intervals. The results of this study showed there was no difference between the two groups in terms of risk of recurrence. The incidence of recurrence or new cancer per follow-up year with MHT was 3%, and with no MHT, it was 4%. Again, this study has limitations, including patient numbers and a short duration, lack of randomization and differences between groups; for example, the MHT group had 10% more node-negative patients than the non-MHT group.

A similar Danish observational cohort study looked at the safety of systemic and vaginal hormone therapy after early breast cancer. The study cohort included postmenopausal women diagnosed with invasive early stage non-metastatic, ER-positive breast cancer over a 7-year period [21]. None of these women received chemotherapy but all patients were allocated to 5 years of tamoxifen, an aromatase inhibitor, both or no endocrine treatment as per guidelines. Among 8461 women who were diagnosed with breast cancer during the period, 1957 used vaginal estrogen therapy (VET) and 133 used menopausal hormone therapy (MHT). The MHT consisted of estrogen replacement therapy (ERT) with or without progesterone (P), as outlined in Table 2. The mode of administration of estrogen varied with the gels, patches and tablets used. The only type of progesterone used was norethisterone. Neither VET nor MHT was associated with a significant increase in recurrence or mortality, the absolute 10-year cumulative risk of recurrence was 19.2% in non MHT users, 15.4% in VET users and 17.1% in users of MHT. Overall, there was no increased risk of recurrence but notably the risk of recurrence (but not mortality) was increased in women using VET whilst also taking an aromatase inhibitor (HR 1.39). Notably, the non-users of menopausal hormonal treatment were older, had larger tumors and were more likely to have lymph node metastasis. The fact this was not a randomized study and the participants did not use a standard regimen of MHT, there are obvious limitations to this study.

Tibolone was used as an MHT regimen for another observational prospective study [22]. The LIBERATE trial was ongoing at the time but no data were published until 2007. In total, 156 postmenopausal women who had been treated for breast cancer and had completed their 5 years of tamoxifen participated in the study. These women were given the option to take tibolone, 52 opted to take 2.5 mg of tibolone daily one month after finishing tamoxifen and 104 women served as controls. In total, 1.9% of the tibolone group developed local recurrence (same breast cancer or contralateral breast cancer) and 2.9% of the control group developed local recurrence, both after a median follow up of 5 years. This showed there was no significant difference between the two groups in terms of breast cancer recurrence. This study also looked at benefits in categories of menopause symptoms: vasomotor, psychological symptoms, somatic and sexual symptoms. Improvement in all four categories was far more evident in the tibolone group than in the control group.

A case–control matched prospective study published by Decker et al. investigated the effect of different types of estrogens given alone as MHT or combined with progestin on the risk of the recurrence of breast cancer. A total of 277 women that were given MHT were followed between 1984 and 2000, and these were matched with appropriate controls [23]. The rate of recurrence was lower or the same in the MHT group for ipsilateral and contralateral breast cancer recurrence compared to the control group. However, rates of metastatic disease and death were higher in the MHT group than the control group. This trial emphasized the effectiveness of MHT on menopausal symptoms, or in their words, ‘estrogen deficiency symptoms’. In the MHT group, 92% of women had relief of vasomotor symptoms, dyspareunia and vaginal dryness was reduced in 89% and mood improved in 88%. It is important to note that the MHT group had more patients with ER-negative breast cancer than the control group.

**Table 2:** This table outlines the odds ratio for recurrence in the treatment group vs. the non-treatment group of the four prospective observational studies discussed. This table also includes the first author of the study and the year of publication. Also outlined is the mean follow-up time in years for each study and the type of MHT agent used. The table compares the sample size, the rate of recurrence of breast cancer per mean follow-up time and the percentage of estrogen receptor-positive tumors in the MHT groups vs. the non-MHT groups.

First Author (Year)	Total, *n*	Recurrence, *n* (%)	Mean Follow Up (Years)	Odds Ratio for Recurrence	ER+, *n* (%)	Type of MHT
Marttunen (2001) [20]			2.5			ERT/ERT + MPA
-MHT	88	7 (8.0%)		0.7	57 (64.7%)	
-No MHT	43	5 (11.6%)			29 (67.4%)	
Decker (2003) [23]			3.7			ERT/ERT + Progestins/ Methyltestosterone
-MHT	271	30 (11%)		0.8	100 (36.9%)	
-No MHT	277	35 (12.6%)			121 (43.6%)	
Dimitrakakis (2005) [22]			5			Tibolone
-MHT	52	1 (1.9%)		0.8	42 (80.7%)	
-No MHT	104	3 (2.9%)			78 (75%)	
Cold (2022) [21]			9.8			ERT/ERT + P/VET
-MHT	2090	127 (6.1%)		0.3	2090 (100%)	
-Non-MHT	6371	1206 (18.9%)			6371 (100%)	

### 3.3. Current Attitudes of MHT in Breast Cancer Survivors

A limitation in evaluating the safety of MHT in breast cancer survivors is the understandable reluctance to take it due to the potential increased risk of recurrence. A feasibility study in the UK between 1994 and 1996 examined the safety of MHT in postmenopausal patients with a history of stage I/II disease [24]. Of 261 eligible women, 38.8% were willing to participate with 100 women randomized to MHT or no MHT. The follow-up period was 6 months. Of those who did receive MHT, 75% of the group continued to take MHT after the trial, reflecting the positive impact of MHT on quality of life, although it also reflected that recruitment for a trial would be challenging.

Vassilopoulou-Sellin et al. published a separate feasibility study, the results of which were gathered during the recruitment period of the RCT discussed above [11]. Of 555 women who were contacted, 136 were ineligible. Of the remaining 418 ER-negative breast cancer survivors remaining, only 17% were willing to take MHT in the setting of a randomized trial. Of those that declined, one-third were concerned about the risk of MHT, one-third were not interested, and the remainder were already on MHT [25]. Interestingly, Wallberg et al. published the results of a questionnaire that was given to 57 participants of the Stockholm trial [26] and 58 non-participants [27]. The study showed that a patient’s decision to accept or decline participation in the Stockholm trial was influenced by their objective risk of breast cancer recurrence.

After the publication of the Women’s Health Initiative [28] and One Million Women study [29], a questionnaire was sent to 469 breast cancer survivors to assess the severity of menopausal symptoms and opinions on starting MHT [30]. Trinh et al. discussed that 76% of women reported vasomotor symptoms and over half of these found the burden of these to be severe in their day-to-day life. In total, 25% of women who answered the questionnaire had been on MHT prior to their breast cancer diagnosis, of whom 80% claimed it improved their quality of life substantially. Data from sixteen observational studies that reported no increased risk of recurrence with MHT were shown to these women. The women were then asked would they be willing to take MHT. In total, 57.9% reported they would be willing to try MHT. Trinh et al. also looked at how willing physicians were to prescribe MHT to breast cancer survivors with severe menopausal symptoms after the WHI was published in a separate study. Again, through a questionnaire, they found only 5% of physicians were willing to prescribe MHT [31]. These studies highlight that whilst the burden of menopause is great, the fear of recurrence is greater.

### 3.4. Strengths and Limitations of Our Study

We purposely included exclusively prospective studies to include the highest quality data available. To the best of our knowledge, a review that includes a combination of prospective trials and observational studies has not been carried out before. This study’s limitations are largely due to the lack of quality data available on this topic, with the majority not meeting target sample sizes, and half of the data coming from observational studies rather than controlled studies and trials ending prematurely. There is obvious variation between the studies discussed in terms of the regimen and dosage of MHT, selection criteria and endpoints. Amongst the studies discussed, there is a lack of consistency with patient characteristics such as nodal positivity or adjuvant hormonal therapy used. It is difficult to confidently analyze and equate these studies when such variations exist.

## 4. Conclusions

There is a paucity of data relating to the impact of MHT on oncological outcomes in breast cancer patients. Interpretation of clinical trials and observational studies is hampered by heterogenicity, small numbers, a short follow up and retrospective nature. Defining a risk of recurrence for breast cancer survivors using MHT is imperative and oncological risk must be weighed against quality of life, as well as long-term benefits of estrogen for osteoporosis and cardiovascular disease. Future studies evaluating low-risk patients with low-dose MHT may be feasible in the future. Randomization of these women in large trials also raises an ethical issue as it is difficult to offer a placebo for a symptomatic climacteric patient willing to receive MHT. An updated opinion on MHT and the burden of menopause from breast cancer survivors would be useful to re-assess the need and feasibility of an RCT. Importantly, in the modern era, there is an opportunity to molecularly risk stratify patients and identify those with genomically low-risk disease (i.e., low oncotype score). With these new developments and safer MHT agents, is now the time to re-examine the role of MHT after breast cancer?

## Figures and Tables

**Table 1:** This is a table that shows the hazard ratio for breast cancer risk of recurrence of MHT compared with non-hormonal replacement therapy in each of the four RCTs discussed. It also outlines the number of patients in each trial, the composition of hormone receptor-positive/negative breast cancers in each trial, the type of MHT used and the median follow up of each trial.

First Author (Trial)	Number of Patients	HR+, *n* (%)	HR−, *n* (%)	Type of MHT	Median Follow Up (Months)	Hazard Ratio (HR)
Vassilopoulou-Sellin	77	0	54 (70.1)	Conjugated estrogen treatment	71	0.52
Holmberg (HABITS)	442	261 (59)	42 (9.5)	Continuous combined or sequential estradiol hemihydrate and norethisterone	48	2.40
Fahlen (STOCKHOLM)	378	216 (57.1)	51 (13.5)	Cyclic estradiol/MPA or estradiol alone	120	1.30
Kenemans (LIBERATE)	3098	2185 (71)	623 (20.1)	Tibolone	36	1.40
